# Introducing and Validating a Minimum Data Set and Core Functionalities for Remote Poststroke Home Monitoring: “A Cross‐Sectional Study”

**DOI:** 10.1002/hsr2.71663

**Published:** 2026-01-06

**Authors:** Mahbubeh Rezazadeh, Azita Yazdani, Amir Ali Ghahremani, Zahra Mahmoudzadeh‐Sagheb

**Affiliations:** ^1^ Student Research Committee, Department of Health Information Management, School of Health Management and Information Sciences Shiraz University of Medical Sciences Shiraz Iran; ^2^ Department of Health Information Management, School of Health Management and Information Sciences, Health Human Resources Research Center Shiraz University of Medical Sciences Shiraz Iran; ^3^ Clinical Education Research Center Shiraz University of Medical Sciences Shiraz Iran; ^4^ Department of Internal Medicine North Khorasan University of Medical Sciences Bojnurd Iran

**Keywords:** data elements, remote patient monitoring, self‐monitoring, stroke, telemedicine

## Abstract

**Background and Aims:**

Stroke is recognized as a significant global health concern. The design and implementation of remote patient monitoring systems necessitate the identification of relevant data elements to effectively address the needs of individuals following a stroke. Identification and validation of these data elements can contribute to the successful design and implementation of remote patient monitoring systems. This study aimed to identify the requirements for a remote monitoring system designed for poststroke patients.

**Methods:**

This descriptive, cross‐sectional study was conducted in three steps, including a literature review, expert panel discussions, and the Delphi technique. The literature was reviewed on electronic databases including the Cochrane Library, Wiley, Scopus, ProQuest, IEEE, PubMed, and Web of Science, using the keywords “stroke,” “secondary stroke,” “remote care,” “telehealth,” “remote patient monitoring,” and “remote monitoring” from 2000 to 2023. Three expert panel sessions were conducted to review and categorize the extracted data elements, with content validity confirmed (CVR 0.89, CVI 0.97). Finally, the Delphi technique involving 20 neurologists was used to validate the finalized data elements and system functionalities.

**Results:**

Thirty‐six studies were selected based on the inclusion criteria. A total of 75 data elements were extracted from the literature review. Finally, 61 data elements in three main categories (demographic information, clinical information, and system functionality) were classified and validated by experts as essential data elements for the design of a remote patient monitoring system for poststroke patients.

**Conclusion:**

The findings of this study encompass a range of fundamental capabilities of remote monitoring systems for poststroke patients. Consequently, this study can guide researchers interested in this field in identifying and selecting the appropriate path for developing a remote patient monitoring application for this group of patients.

## 1 Introduction

Stroke is a leading cause of death and disability globally, imposing significant economic and social burdens. According to the World Health Organization's 2024 report, ~15 million people worldwide experience a stroke annually, with nearly 5 million experiencing permanent disability. Beyond the physical and cognitive impairments, stroke significantly impacts patients' social, economic, and psychological well‐being. Consequently, long‐term rehabilitation and management of poststroke patients are crucial [1]. Considering the rising incidence of stroke, particularly among older populations, there is a growing need for effective and sustainable strategies to manage and monitor patients during the poststroke period. Despite significant advancements in diagnosis and treatment, there remains a need for effective preventive strategies and sustained rehabilitation interventions [2, 3]. Despite significant progress in acute stroke management and secondary prevention strategies, recurrent strokes remain a major challenge. Recurrent strokes account for 25%–30% of all strokes and are more debilitating, lethal, and costly than the initial stroke. Therefore, optimal poststroke care requires continuous monitoring of various health parameters, including blood pressure, medication adherence, physical activity, and cognitive function, to prevent recurrent strokes and achieve better long‐term outcomes [4]. Current stroke prevention strategies include medications like antithrombotic, and interventions aimed at modifying vascular risk factors [5]. However, some patients consistently fail to follow medical advice, and medication adherence after stroke is very poor [6, 7]. Optimizing vascular risk factors is often difficult and complex, as stroke patients may have diverse demographic characteristics and comorbidities [8, 9]. Since poststroke management is a lifelong process, remote patient monitoring systems offer a promising avenue for improving poststroke care. Remote patient monitoring utilizes technology to collect, transmit, and analyze health data outside of clinical settings. Implementing remote patient monitoring systems is essential for controlling risk factors, ensuring a more comprehensive and systematic secondary prevention strategy, and improving ongoing coordination among specialists, patients, and caregivers. For stroke patients, these systems can play a crucial role in managing vascular risk factors, ensuring medication adherence, monitoring rehabilitation progress, improving coordination among healthcare providers, patients, and caregivers, controlling blood pressure, promoting patient engagement in treatment, and raising stroke awareness [10].

Remote patient monitoring systems involve the collection, transmission, analysis, and sharing of patients' personal health data with their care team from outside of healthcare settings [11, 12, 13]. In fact, remote patient monitoring is one of the most important programs for stroke patient care in developed countries. The primary goal of remote patient monitoring is to prevent prolonged hospital stays and reduce treatment costs, as well as helping patients improve their self‐care skills and encourage more active participation in their own care through real‐time feedback [14, 15]. These systems can help with early detection of complications, preventing readmissions, and improving patients' quality of life. Through continuous, noninvasive monitoring, these systems enable healthcare providers to intervene more quickly and effectively when needed, thus reducing readmission rates and associated costs. Benefits of remote patient monitoring include managing the patient's condition, enabling continuous or intermittent patient monitoring, preventing disease exacerbation and premature death, reducing hospital costs, decreasing hospitalizations, and providing access to detailed patient records [16, 17].

Effective design of remote patient monitoring systems requires a thorough understanding of the needs of patients, healthcare providers, and the healthcare system. These needs form the foundation for system design. A comprehensive identification of essential needs and data elements ensures that the designed system is appropriate, efficient, and ultimately beneficial for improving healthcare and reducing costs. This process involves identifying critical variables, required monitoring parameters, and methods for data collection, storage, and analysis. Neglecting this stage leads to inefficient systems, a lack of alignment with real needs, and ultimately, a failure to improve patient health outcomes [10, 16].

Based on the reviewed literature, essential system requirements for remote patient monitoring systems for poststroke were identified and validated for the first time in this study. Therefore, this study aimed to identify and determine the fundamental requirements and functionality of remote patient monitoring systems for stroke patients, aiming to design a comprehensive system based on patient needs.

## 2 Methods

This descriptive cross‐sectional study was conducted in 2023–2024 to identify, classify, and validate the data elements and system's functionality required to design a remote patient monitoring system for poststroke patients. In this study, data elements refer to the specific information captured by the system (e.g., vital signs, medication records), whereas system functionalities denote the system's operational features or actions (e.g., data visualization, alerts, or communication tools).

**Figure 1:**
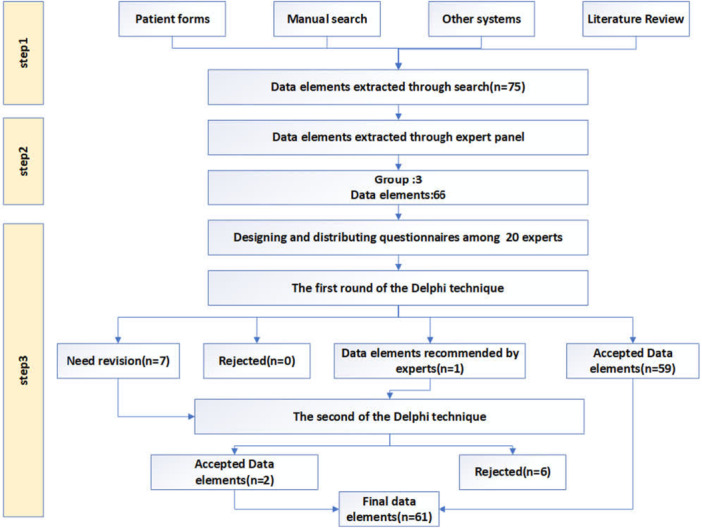
Flowchart of data elements determination.

The research method included conducting the literature review, implementing the expert panel, and employing the Delphi technique (Figure 1).

The research steps were as follows.

### 2.1 Step 1: Literature Review

In this step, the research literature was reviewed from January 1, 2000 to October 31, 2023. Seven databases (Cochrane Library, Wiley, Scopus, ProQuest, IEEE, PubMed, and Web of Science) were reviewed based on keywords such as “stroke,” “secondary stroke,” “remote care,” “telehealth,” “remote patient monitoring,” and “remote monitoring.” To extract the relevant studies, the titles and abstracts of studies were searched for by using a combination of keywords and search strategies shown in Table 1. Additionally, a manual search process was implemented on patient forms, similar systems, and the Google Scholar search engine (by analyzing the first 10 pages). The detailed search strings used for each database are provided in Appendix 1.

**Table 1:** The search strategy.

#1	(Telemedicine OR Telehealth OR Telecare OR Telemonitoring OR “Remote monitoring” OR “Remote assessments” OR “Remote management” OR “Remote monitoring system” OR “Remote care” OR “Patient telemonitoring” OR “Remote patient monitoring” OR “Self‐monitoring” OR Monitoring OR MHealth OR “Mobile apps” OR “MHealth applications”)
#2	(Stroke OR “Post‐stroke” OR Poststroke OR “Stroke care” OR “Secondary stroke after stroke”)
#3	#1 AND #2
Limit	Language: English; Time: 2000–2023

*Note:* The Boolean operator used between #1 and #2 is **AND**. The detailed database‐specific search strings, including Boolean logic and field specifications, are provided in Appendix 1.

### 2.2 Inclusion and Exclusion Criteria

The inclusion criteria encompassed the papers published in English, access to the full texts of papers, and the presence of data elements and clinical information on stroke and poststroke patients. Included studies were original research articles published in English with full‐text availability, focusing on data elements and clinical information related to poststroke patients monitored remotely. Excluded were conference papers, letters to the editor, commentaries, short communications, studies not involving remote patient monitoring, and those lacking clear information about data elements or system functionalities.

**Figure 2:**
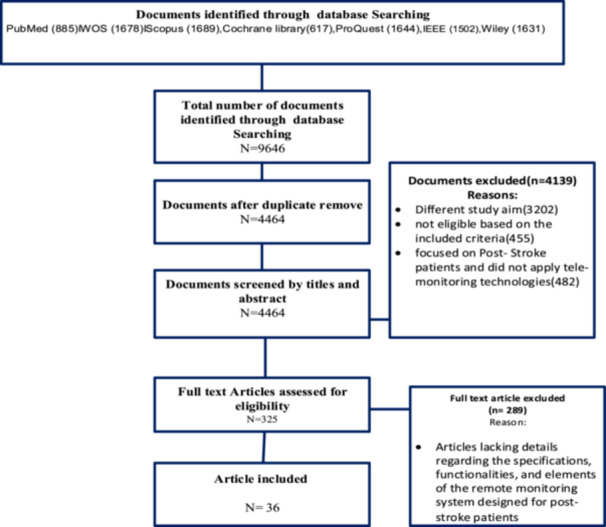
PRISMA 2020 flow diagram showing the study selection process for inclusion in the data elements extraction phase.

The exclusion criteria included conference papers, papers that did not mention the design and development of remote patient monitoring systems, and papers that did not contain clear information on data elements. Furthermore, letters to the editors and conference abstracts were excluded (Figure 2).

### 2.3 Selection and Classification of Papers

As per the search strategy, 9646 papers were retrieved from 7 databases and entered into Mendeley software, version 2.118.0. Additionally, 1689 studies were retrieved from Scopus, 1678 from Web of Science, 1644 from ProQuest, 1631 from Wiley, 1502 from IEEE, 885 from PubMed, and 617 from the Cochrane Library. However, 5182 papers were excluded after duplicates were reviewed. The exclusion of papers was based on the exclusion criteria. The titles, abstracts, and keywords of the remaining 4464 articles were then carefully analyzed. Finally, 36 papers were included in the study. The full texts of these papers were reviewed to extract the data elements required for the design and implementation of a remote monitoring system for poststroke patients. Furthermore, seven similar systems were searched and reviewed to extract data elements. A data extraction form was used to collect the data. This form was validated by a medical informatics specialist, three neurologists, and a health information management specialist. The checklist included fields for data elements and system functionality (Appendix 2). For instance, items such as “blood pressure monitoring,” “medication adherence,” and “physical activity tracking” were repeatedly identified across multiple mHealth stroke studies and were therefore included in the initial Delphi questionnaire. All retrieved articles were imported into Mendeley (version 2.118.0), where duplicates were removed using the software's deduplication feature. A manual check was also performed to ensure all duplicates were excluded.

### 2.4 Step 2: Expert Panel

In the first step, the data elements and system functionality were extracted from various sources. Therefore, three expert panel sessions were held to review, classify, and tailor the initial drafts of data elements and functionality of the system to the specific needs of stroke patients and to prepare a checklist. Experts were selected based on inclusion criteria such as at least 5 years of professional experience, relevant academic background, and expertise in stroke care or health IT. Those lacking relevant experience or unavailable for participation were excluded. All panel members were faculty members with relevant expertise, and consensus during the sessions was achieved through discussion and verbal agreement. To ensure consistency among the experts, each member independently reviewed and classified the extracted data elements before the panel meetings, and any discrepancies were discussed and resolved through consensus with the research team. The list of data elements and functionality extracted from the literature review was discussed in the first session. Subsequent sessions were held with the experts to review, adapt, and classify the initial drafts of data elements and system requirements. At this step, the experts were provided with only a list of data elements. They were also asked to express their opinions on the approval or rejection of each data element and its placement in the relevant dimension. Finally, the data elements and system requirements checklist were designed. The face validity of the checklist was confirmed using the opinions of a neurologist, two medical informatics specialists, and two health information management experts (*n* = 5). The content validity of the checklist was calculated using the content validity ratio (CVR) and the content validity index (CVI). For this purpose, three medical informatics specialists, three information management specialists, and four neurologists completed the checklist. According to Lawshe's criterion for CVR, when the focus group consists of 10 members, the minimum acceptable value for each item is 0.62.37. CVR was confirmed with a value of 0.89. Also, CVI was confirmed with a value of 0.97. In total, 10 experts (3 medical informatics specialists, 3 health information management experts, and 4 neurologists) participated in the CVR and CVI assessments. Table 2 summarizes the specialties, affiliations, and academic positions of the expert panel members to demonstrate representativeness

**Table 2:** Summary of expert panel composition.

Specialty/discipline	Affiliation	Academic position/role
Neurology (*n* = 4)	University Hospitals, Stroke Research Centers	Associate/assistant professors
Medical informatics (*n* = 3)	School of Health Management and Information Sciences	Associate professors/researchers
Health information management (*n* = 3)	School of Health Management and Information Sciences	Associate professors/researchers

Consensus during the expert panel sessions was reached through discussion and verbal agreement among members. Each expert independently reviewed the proposed data elements before the meetings, and discrepancies were resolved through group discussion until full consensus was achieved.

Five experts independently reviewed and classified the extracted data elements into these predefined categories. Interrater reliability among the experts was assessed using Cohen's *κ* coefficient, which showed excellent agreement (*κ* = 0.82) [18]. Any discrepancies were discussed and resolved through consensus with the research team to ensure consistency in classification

The minimum acceptable CVR value of 0.62 was selected based on Lawshe's table for a panel size of ten raters, consistent with our study [19]. The reliability of the checklist was evaluated by Cronbach's *α* and confirmed with a value of 0.94.

### 2.5 Step 3: Delphi Technique

The checklist consisted of two parts: demographic information for the experts (5 items) and essential data elements (66 items), which were classified under 3 main categories: demographics (22 items), clinical information (25 items), and system functionality (19 items). Moreover, a 5‐point Likert scale (*totally unnecessary* = 1, *unnecessary* = 2, *no opinion* = 3, *necessary* = 4, and *totally necessary* = 5) was employed to score each element. The Delphi technique was adopted in two rounds to reach a consensus, validate the drafted checklist of data elements and functionality, and receive suggestions from experts. This study was designed and reported in accordance with the Conducting and Reporting of Delphi Studies (CREDES) guidelines to ensure methodological transparency and reproducibility. The Delphi technique is an appropriate method in contexts where statistical model‐based evidence is unavailable, knowledge is uncertain and incomplete, and human expert judgment is better than individual judgment [20]. In most Delphi studies, 10–30 experts are invited to complete a checklist. Therefore, in this study, purposive sampling was used to select 20 neurologists to review the draft data elements and functionality [21]. The sample size of 20 experts was determined based on the common practice in Delphi studies to balance diversity of opinions and feasibility, ensuring methodological rigor while considering expert availability. Informed consent was obtained orally from the participants before participating in the study. The following inclusion criteria were used for the participants:

– Employment as a member of the faculty of medical sciences universities.
– Having more than 5 years of work experience in the field of neurology.
– Participants with < 5 years of experience or those unwilling to participate were excluded from the study.

The checklist was designed electronically and emailed to the experts along with a brief explanation of the study's purpose. The two Delphi rounds were conducted over a 6‐week period (3 weeks/round). All 20 invited experts completed both rounds of the Delphi process, resulting in a 100% response and retention rate. Although no attrition occurred, reminder emails were sent before each round to ensure full participation and minimize potential nonresponses. After the first round, summarized feedback including median scores and anonymized comments was provided to participants to help guide their responses in the second round. Changes in item scores between rounds were analyzed, and any disagreements were resolved through further discussion and consensus among the experts. Additionally, to identify other important data elements, an open‐ended question was included at the end of each section titled other data elements. After experts completed the checklists in the first and second rounds of the Delphi technique, the data were entered into SPSS version 23 (IBM). Frequency and mean scores for each data element were then analyzed and reported. Descriptive statistics, including frequency, percentage, mean, and standard deviation (SD), were used to summarize experts' ratings and determine consensus levels. No inferential or hypothesis tests were performed, as the Delphi method focuses on achieving expert agreement rather than statistical significance [19, 20]. Statistical reporting followed the general principles of the Statistical Analyses and Methods in the Published Literature (SAMPL) guidelines and standard references for Delphi methodology. Decisions were made to remove data elements with an agreement level below 50% (mean score < 2.5) in the first round of the Delphi process. Data elements with agreement levels between 50% and 75% (mean score 2.5–3.75) were retained for the second round, and those with agreement levels above 75% (mean score > 3.75) were considered final. These cutoff thresholds were based on methodological recommendations by Hsu and Sandford [19, 21], who indicated that mean scores above 3.75 represent strong consensus for acceptance, whereas scores below 2.5 reflect disagreement for rejection. A total of 66 items were initially included in the Delphi process, and 61 items remained after two rounds.

## 3 Results

This study initially identified 9646 articles from the searched databases. After removing duplicates, 4464 articles remained for title and abstract screening. Subsequently, 4139 articles were excluded due to: irrelevant study objectives (*n* = 3202), not meeting inclusion criteria (*n* = 455), not focused on poststroke patients or remote monitoring technologies (*n* = 482). During full‐text review, 325 articles were assessed for eligibility, and 289 were excluded for lacking sufficient details on specifications, functionalities, or data elements of remote monitoring systems designed for poststroke patients. Finally, 36 articles were included for data extraction (Figure 2).

These studies were published between 2012 and 2023, originating from multiple countries across North America, Europe, and Asia. The largest number of studies were from the United States, followed by China and Canada. Most papers focused on the design, implementation, or evaluation of remote monitoring systems and mHealth applications for poststroke patient care, reflecting the growing international interest in technology‐supported stroke management. This process resulted in the identification of 75 data elements and system functionality found in existing remote patient monitoring systems for poststroke patients. Among the 66 initial items evaluated, 5 items fell below the 0.62 threshold and were excluded prior to the Delphi rounds. The remaining 61 items met the minimum CVR and CVI criteria and entered the Delphi process. Therefore, the final list of 61 data elements was ranked according to their mean scores in the second Delphi round to reflect their relative priority from the experts' perspective.

Table 3 presents the demographic characteristics of the experts. Male participants accounted for 65% of the sample, which was higher than the proportion of female participants. The largest age group comprised experts aged 40–49 years.

**Table 3:** Frequency of demographic information of study participants.

Variables		Frequency	Percentage
Gender	Male	13	65%
	Female	7	35%
Age (years)	< 40	4	20%
	40–49	11	55%
	≥ 50	5	25%
Level of education	Fellowship (subspecialty‐trained)	1	5%
	Specialist (board‐certified)	19	95%

As shown in Figure 1, the literature review identified 75 key data elements and system functionalities extracted from peer‐reviewed articles, similar systems, and patient record forms. During the expert panel sessions, duplicates and irrelevant items were removed, resulting in 66 elements categorized into 3 main groups: demographic information (22 items), clinical information (25 items), and system functionalities (19 items).

According to the first round of the Delphi technique, 59 elements achieved consensus (mean > 3.75), while 7 items were carried forward to the second round (mean = 2.5–3.75). In the second round, one additional item—“evaluation of the patient's progress”—was validated with an agreement above 75%, and six items were excluded (mean < 2.5). The questionnaire was distributed electronically to 20 neurologists, all of whom completed both rounds, yielding a 100% response rate. The mean scores from the Delphi rounds were used to describe experts' perceptions of importance rather than to establish a formal ranking or weighting (Table 4).

**Table 4:** Categories of essential data elements.

Category	The number of data elements	First round Delphi			Second round Delphi			The final number of data elements
		< 50%	50%–75%	> 75%	< 50%	50%–75%	> 75%	
Demographic information	22	0	7	15	6	0	1	16
Clinical information	25	0	0	25	0	0	0	25
Functionality of the system	19	0	0	19	0	0	1	20

Regarding demographic data, the survey results showed strong consensus (over 75% agreement) among experts on the inclusion of national ID, first and last name, age, gender, and BMI of patients in the remote patient monitoring system. However, the Delphi technique analysis of the second round revealed that six elements of patient‐related data—RH (Rhesus) blood type (45%), number of family members (37%), insurance status (44%), age of the patient care team (24%), gender of the patient care team (27%), and education level of the patient care team (37%)—were excluded due to average scores below 2.5 (representing < 50% agreement). Conversely, marital status received strong support (88%) (Table 5).

**Table 5:** Results from the Delphi technique to determine the level of necessity of demographic data.

Data element	Category	Decision (Round 1)	Mean (SD) (Round 1)	Decision (Round 2)	Mean (SD) (Round 2)
First name, last name	Demographic information	Accepted	5 (0.000)	—	—
Father's name	Demographic information	Accepted	4.4 (1.095)	—	—
National code	Demographic information	Accepted	5 (0.000)	—	—
Gender	Demographic information	Accepted	5 (0.000)	—	—
Age	Demographic information	Accepted	5 (0.000)	—	—
Weight	Demographic information	Accepted	4.75 (0.639)	—	—
Height	Demographic information	Accepted	4.5 (1.000)	—	—
BMI	Demographic information	Accepted	5 (0.000)	—	—
RH (Rhesus) blood type	Demographic information	Getting to the second round	2.75 (0.716)	Decline	2.25 (0.786)
Blood group type	Demographic information	Accepted	4.5 (0.889)	—	—
Marital status	Demographic information	Getting to the second round	3.5 (1.051)	Accepted	4.4 (0.503)
Level of education	Demographic information	Accepted	4.5 (0.946)	—	—
Phone number	Demographic information	Accepted	5 (0.000)	—	—
Address	Demographic information	Accepted	4.5 (0.889)	—	—
Number of family members	Demographic information	Getting to the second round	2.55 (0.759)	Decline	1.85 (0.671)
Occupation	Demographic information	Accepted	4.55 (0.918)	—	—
Insurance status	Demographic information	Getting to the second round	2.7 (0.801)	Decline	2.2 (0.951)
First name, last name of provider	Demographic information	Accepted	4.00 (1.729)	—	—
Age of provider	Demographic information	Getting to the second round	2.75 (0.639)	Decline	1.20 (0.523)
Gender of provider	Demographic information	Getting to the second round	2.6 (0.754)	Decline	1.35 (0.489)
Level of education of provider	Demographic information	Getting to the second round	3.05 (0.605)	—	—
Specialized field of provider	Demographic information	Accepted	4.66 (1.460)	—	—

The data elements related to clinical data were accepted with an agreement of over 90%. And the items of current medications, blood pressure, heart rate, type of stroke, date of stroke, National Institutes of Health Stroke Scale (NIHSS) score, and CT scan images obtained the highest score and agreement of 100% (Table 6).

**Table 6:** Results from the Delphi technique to determine the level of clinical data necessity.

Data element	Category	Decision (Round 1)	Mean (SD) (Round 1)	Decision (Round 2)	Mean (SD) (Round 2)
Past medical history	Clinical information of patient medical history	Accepted	4.9 (0.308)	—	—
Patient symptoms (paresis, dizziness, blurred vision, swallowing disorder)	Clinical information of patient medical history	Accepted	4.95 (0.224)	—	—
History of drug use and current medications	Clinical information of patient medical history	Accepted	5 (0.000)	—	—
Smoking status	Clinical information of patient medical history	Accepted	4.85 (0.366)	—	—
Alcohol consumption status	Clinical information of patient medical history	Accepted	4.95 (0.224)	—	—
Blood pressure	Clinical information of vital sign	Accepted	5 (0.000)	—	—
Heart rate	Clinical information of vital sign	Accepted	5 (0.000)	—	—
Body temperature	Clinical information of vital sign	Accepted	4.85 (0.489)	—	—
Respiratory rate	Clinical information of vital sign	Accepted	4.75 (0.639)	—	—
Type of stroke (ischemic, hemorrhagic)	Clinical information of patient stroke data	Accepted	5 (0.000)	—	—
Date of stroke occurrence	Clinical information of patient stroke data	Accepted	5 (0.000)	—	—
Location of brain lesion	Clinical information of patient stroke data	Accepted	4.95 (0.224)	—	—
History of NIHSS score	Clinical information of patient stroke data	Accepted	5 (0.000)	—	—
Number of strokes	Clinical information of patient stroke data	Accepted	4.9 (0.308)	—	—
Family history of stroke	Clinical information of patient stroke data	Accepted	4.8 (0.523)	—	—
CBC	Clinical information of paraclinical data	Accepted	4.9 (0.308)	—	—
Lipid profile	Clinical information of paraclinical data	Accepted	4.75 (0.550)	—	—
Vitamin D3	Clinical information of paraclinical data	Accepted	4.7 (0.657)	—	—
Fasting blood sugar	Clinical information of paraclinical data	Accepted	4.85 (0.366)	—	—
CT scan	Clinical information of paraclinical data	Accepted	5 (0.000)	—	—
MRI	Clinical information of paraclinical data	Accepted	4.9 (0.308)	—	—
Sonography	Clinical information of paraclinical data	Accepted	4.8 (0.523)	—	—
Drug therapy	Clinical information of treatment procedure	Accepted	4.85 (0.366)	—	—
Rehabilitation treatment (occupational therapy, physiotherapy)	Clinical information of treatment procedure	Accepted	4.75 (0.550)	—	—
Surgical treatment (angiography, angioplasty, etc.)	Clinical information of treatment procedure	Accepted	4.65 (0.671)	—	—

Regarding the system functionality items, almost all experts agreed or strongly agreed with all items and achieved an agreement of over 90%. And the items of providing timely warnings of abnormal clinical and vital signs (blood pressure, temperature, heart rate, respiration, weight), reminding when to take medication, reminding when to do tests, next visit and rehabilitation measures, answering patients' common questions, educating about risk factors and educating on immediate actions in emergencies achieved the highest score and 100% agreement. Also, in the open questions or other data elements section, experts suggested evaluating the patient's progress. This was validated due to achieving an average of over 75% in the second round of the Delphi technique (Table 7).

**Table 7:** Results from the Delphi technique to determine the level of necessity of system functionality items.

Data element	Category	Decision (Round 1)	Mean (SD) (Round 1)	Decision (Round 2)	Mean (SD) (Round 2)
Displaying reports of clinical data	Functionality of system	Accepted	4.85 (0.366)	—	—
Providing alerts for abnormal clinical and vital signs (blood pressure, temperature, heart rate, respiration, weight)	Functionality of system	Accepted	5 (0.000)	—	—
Medication reminder	Functionality of system	Accepted	4.7 (0.657)	—	—
Reminder for doing laboratory tests, follow‐up visits, and rehabilitation activities	Functionality of system	Accepted	5 (0.000)	—	—
Virtual communication with a doctor (chat forum)	Functionality of system	Accepted	4.95 (0.224)	—	—
Virtual communication with a doctor (video call)	Functionality of system	Accepted	4.1 (1.089)	—	—
Responding to frequently asked questions by patients	Functionality of system	Accepted	5 (0.000)	—	—
Patient status dashboard	Functionality of system	Accepted	4.75 (0.550)	—	—
Evaluation of the progress of patient	Functionality of system	Recommended by experts in the first round	—	Accepted	4.5 (0.946)
Patient education: basic information about stroke (definition and symptoms)	Functionality of system	Accepted	4.85 (0.826)	—	—
Patient education: information about the treatment of stroke	Functionality of system	Accepted	4.65 (0.745)	—	—
Patient education: information about the complications of stroke	Functionality of system	Accepted	4.65 (0.946)	—	—
Patient education: drug information and the importance of medication use	Functionality of system	Accepted	4.8 (0.523)	—	—
Patient education: information about blood pressure and its importance in control	Functionality of system	Accepted	4.9 (0.308)	—	—
Patient education: information about preventing recurrent stroke	Functionality of system	Accepted	4.95 (0.224)	—	—
Patient education: Information about poststroke depression and the importance of its treatment	Functionality of system	Accepted	4.85 (0.489)	—	—
Patient education: information about nutrition	Functionality of system	Accepted	4.8 (0.523)	—	—
Patient education: information about rehabilitation and exercise	Functionality of system	Accepted	4.95 (0.224)	—	—
Patient education: information about risk factors	Functionality of system	Accepted	5 (0.000)	—	—
Patient education: information about immediate actions in emergency situations	Functionality of system	Accepted	5 (0.000)	—	—

*Note:* The item “Evaluation of the progress of patient” was not part of the original list but was added to the second round based on expert recommendations during the first round of the Delphi process.

## 4 Discussion

This study extracted and validated essential data elements required for designing a remote patient monitoring system for poststroke patients. The needs assessment step is a critical activity in the software development lifecycle, and the quality of the requirements directly impacts project success. During this phase, user and system requirements are carefully identified, analyzed, and documented. These requirements form the foundation for system design. The specification phase serves as a roadmap for software development, and neglecting it significantly increases the risk of project failure [22]. In the present study, relevant data elements and functionality for designing the remote patient monitoring system for poststroke patients were extracted following a literature review. In the second stage, expert panel meetings were held. Redundant and unnecessary elements were removed, and then 66 data elements were categorized into three main groups: demographic data, clinical data, and system functionality. Following the completion of the first round of the Delphi technique, the data elements including Rh blood type, number of family members, insurance status, marital status, age of the patient care team, gender of the patient care team, and education level of the patient care team did not receive sufficient scores. For further evaluation, they were included in the second round of the Delphi technique, in which six of the data elements were eliminated by the experts, and only marital status was accepted. Additionally, in the open‐ended questions section or other data elements, the experts suggested a data element related to the ability to evaluate patient progress. This data element was validated due to achieving an average score above 75% in the second round of the Delphi technique. In this study, experts discussed the importance of including cognitive, psychological, and functional aspects in the remote monitoring process. During the Delphi rounds, these dimensions were conceptually integrated into the newly added item “Evaluation of the patient's progress,” which covers elements such as cognitive status, mood, and quality of life in a comprehensive and practical manner suitable for system design.

These results highlight the importance of these data elements in the design and implementation of a remote patient monitoring system for poststroke patients. Caregivers play a vital role in the recovery process and overall health of poststroke patients [23]. These are involved not only in physical care, medication management, and providing emotional support to patients, but also in communication and health monitoring [24]. Their responsibilities include accompanying patients to medical appointments, communicating the patients' needs and concerns to healthcare providers, and ensuring that the patient receives appropriate care and treatment [25]. The health of poststroke patients is usually monitored by caregivers who look for any signs of complications or changes in their condition [26, 27]. These important roles may be the reason why patient caregiver data was considered essential the panel of experts categorized the data. In this study, personal identification data and socioeconomic data are subsets of demographic information. Clinical data were categorized into five subgroups: the patient's medical history, vital signs, paraclinical data, stroke‐related information, and treatment methods.

Blood pressure was consistently recorded as a vital sign in all the reviewed studies. Elevated blood pressure is a significant risk factor for both initial and recurrent strokes, highlighting its crucial importance [28]. The 2014 American Heart Association and Stroke Association guidelines underscore that effective blood pressure management is arguably the most crucial preventative measure against stroke [24]. Furthermore, patients with a history of stroke are at higher risk for secondary strokes, which can be mitigated by long‐term blood pressure reduction [29, 30]. This underscores the importance of controlling blood pressure in this population. Remote patient monitoring is a potentially effective method for achieving and maintaining blood pressure control [31, 32, 33].

Within the subgroup of information related to the patient's stroke, the NIHSS is one of the most important data elements mentioned in several studies [9, 27, 34, 35, 36, 37, 38, 39, 40, 41]. The NIHSS is a quantitative and qualitative tool used to assess the severity of stroke‐related neurological deficits. It helps monitor changes over time and informs medical decisions. Essentially, it provides a numerical representation of the patient's neurological impairment [42]. The NIHSS score is crucial in assessing stroke severity, guiding treatment decisions, and predicting patient outcomes [43, 44, 45]. Furthermore, this score is significant in remote patient monitoring for poststroke patients because it allows for: remote assessment of stroke severity, early detection of deterioration, personalized treatment planning, efficient resource allocation, high long‐term follow‐up, data‐driven decision making, and enhanced communication^45^, ^46^, ^47^].

The system includes functionalities already established in other studies, such as alerts for blood pressure, blood glucose, weight, and medication reminders. It also monitors vital signs based on self‐reported patient data [2, 9, 27, 33, 35, 36, 37, 38, 40, 48, 49, 50, 51, 52, 53, 54, 55, 56, 57, 58, 59, 60, 61, 62, 63, 64, 65, 66, 67, 68, 69]. One reason why the use of these functions is important is that the major focus is on managing modifiable risk factors for stroke. This implies that the functions in question are likely designed to identify, track, and/or intervene on those risk factors [70]. Modifiable risk factors are of high importance, as interventional strategies aimed at reducing these factors can subsequently reduce the risk of stroke. These factors include high blood pressure, diabetes, dyslipidemia, atrial fibrillation and other heart conditions, carotid artery stenosis, smoking, poor diet, physical inactivity, and obesity. Addressing these factors through lifestyle changes and/or medication can significantly impact a person's stroke risk [71].

Therefore, given the widespread use of mobile phone technology [72], mHealth represents a valuable and discreet tool for managing stroke populations with highly modifiable risk factors [73]. This is particularly relevant because mHealth interventions can potentially improve adherence to lifestyle changes and medication regimens, which are crucial for reducing stroke risk [50].

Patient education is critical for successful stroke recovery and the prevention of future strokes. Remote patient monitoring systems should incorporate comprehensive education tailored to individual needs, addressing the significant unmet educational demands of stroke survivors and caregivers regarding stroke management, prevention, treatment, and functional recovery [74, 75]. Daily care education interventions for stroke patients reduce caregiver burden in physical, psychological, and social domains [76, 77, 78]. Therefore, patient education is considered a functionality of the system.

Features such as video conferencing and a patient status dashboard were identified as essential components of a remote monitoring system. However, implementing video communication requires consideration of several practical and regulatory issues to ensure safe and effective use. These include protecting patient privacy through secure and encrypted data transmission, ensuring sufficient bandwidth and reliable connectivity, addressing digital literacy challenges among stroke survivors, and complying with legal frameworks such as HIPAA^1^ and GDPR^2^. These factors must be addressed during system design to ensure that video‐based interactions are both usable and clinically safe [79, 80, 81]. A patient status dashboard conceptually provides an overview of key patient data, including vital signs (blood pressure, heart rate, temperature, and weight), medication intake records, alerts, and rehabilitation progress, displayed through unified charts and tables. These conceptual recommendations highlight the role of these features in supporting remote patient–doctor interaction, enhancing patient monitoring, and improving care quality and process efficiency. These findings are consistent with previous studies [82].

Several previous studies have proposed mHealth‐based systems for poststroke management, such as Seo et al. [9], Sakakibara et al. [35], Kim et al. [51], and Zhang et al. [63]. These systems primarily focused on monitoring specific risk factors such as blood pressure control, medication adherence, or rehabilitation exercises using mobile or web‐based applications. While these studies successfully demonstrated feasibility and patient engagement, their data elements were often limited to a narrow set of clinical or behavioral parameters. In contrast, the present study provides a more comprehensive, consensus‐based minimum data set that encompasses demographic, clinical, and system functionality dimensions validated by a panel of neurologists. Unlike prior mHealth tools, which were typically developed for single‐function monitoring, our proposed data set serves as a structured foundation for developing integrated remote monitoring systems that combine physiological data, educational components, and automated alerts tailored to poststroke care needs.

One of the system's key functionalities is responding to frequently asked questions by patients, aimed at supporting patient education and self‐management. It provides structured, evidence‐based answers to common nonemergency inquiries, such as lifestyle recommendations, medication schedules, or rehabilitation exercises. In its initial design, the system is semi‐automated, presenting predefined, clinically validated information while directing urgent or complex medical queries to clinicians to ensure patient safety. Although this feature offers clear educational benefits, several challenges remain. These include response accuracy, risk of misdiagnosis, algorithmic bias, protection of sensitive data, and lack of human empathy. Future deployment of AI‐driven enhancements will require careful evaluation of technical feasibility, data security, patient privacy, and algorithmic transparency to prevent misinformation and maintain medical accountability. Previous studies have reported risks associated with AI‐based health communication, such as guidance errors, data breaches, and regulatory noncompliance [83, 84]. Therefore, while the functionality may enhance patient education and self‐management, future research should focus on rigorous technical, ethical, and safety assessments, alongside validation with end‐user feedback, clinical outcomes, and benchmarking against existing systems.

### 4.1 Limitations and Future Works

This study has several limitations. Participation was restricted to local neurologists, limiting both multidisciplinary input and international perspectives, which may affect the generalizability of the findings. Other stakeholders, including patients, caregivers, nurses, and technologists, were not involved, potentially restricting the diversity of viewpoints. The literature review was limited to English‐language, peer‐reviewed publications, excluding conference papers and abstracts. While this ensured methodological completeness, emerging innovations in mHealth and telemonitoring may have been overlooked. No formal quality appraisal or prioritization of the extracted data elements was performed, as the primary aim was to identify relevant functional and nonfunctional requirements for a remote patient monitoring system for poststroke patients during the conceptual design phase. This study focused on conceptual design rather than system implementation. Empirical validation, usability testing, and clinical outcome evaluation were not conducted. Consequently, reliance on expert consensus introduces potential methodological limitations, including confirmation bias, groupthink, or dominance of senior participants. Although core functionalities such as blood pressure monitoring, patient education, and caregiver involvement have broad clinical relevance, the findings should be interpreted cautiously due to the single‐national context of the expert panel. Future research should adopt multidisciplinary, international, and user‐centered approaches to enhance external validity, guide implementation, and assess real‐world feasibility and clinical effectiveness.

## 5 Conclusion

Stroke is a chronic condition that requires continuous and long‐term management. Implementing a remote patient monitoring system is therefore essential for improving patient care and clinical outcomes. Such systems have the potential to transform poststroke management by enabling ongoing monitoring, early detection of complications, and timely clinical interventions. By incorporating the validated data elements identified in this study, developers can design comprehensive, patient‐centered platforms that enhance care quality, empower patients, and support clinicians in decision‐making. The consensus‐based minimum data set and core functionalities established in this research highlight key priorities for system design and development. Integrating these validated components, such as vital sign monitoring, medication reminders, and educational features, can improve adherence, strengthen care coordination, and reduce the risk of recurrent stroke. Overall, these findings provide a solid, evidence‐based foundation for future research and innovation aimed at developing user‐centered, technology‐driven solutions that ensure continuity of care and promote better long‐term outcomes for stroke survivors.

## Author Contributions

**Mahbubeh Rezazadeh:** conceptualization, investigation, methodology, validation, software, formal analysis, data curation, visualization, writing – original draft, writing – review and editing. **Azita Yazdani:** conceptualization, methodology, data curation, validation, writing – review and editing, writing – original draft. **Amir Ali Ghahremani:** data curation, validation, formal analysis, writing – original draft, writing – review and editing. **Zahra Mahmoudzadeh‐Sagheb:** conceptualization, investigation, writing – original draft, methodology, validation, writing – review and editing, supervision, project administration.

## Disclosure

The lead author Zahra Mahmoudzadeh‐Sagheb affirms that this manuscript is an honest, accurate, and transparent account of the study being reported; that no important aspects of the study have been omitted; and that any discrepancies from the study as planned (and, if relevant, registered) have been explained.

## Ethics Statement

The study was approved by the Ethics Review Board of Shiraz University of Medical Sciences (Ethical Code: IR.SUMS.NUMIMG.REC.1401.086). The study was performed in compliance with this institutional guideline, the ethical guidelines for clinical research of the Iranian government.

## Consent

Participants gave their informed consent to participate.

## Conflicts of Interest

The authors declare no conflicts of interest.

## Data Availability

The data that support the findings of this study are not publicly available due to privacy and confidentiality restrictions related to expert participants. However, aggregated data and summarized results are available within the article. Additional information may be obtained from the corresponding author upon reasonable request.

## Endnotes

1 Health Insurance Portability and Accountability Act.

2 General Data Protection Regulation.

## Appendix 1 (Search String)

**Table:**

Database	Search string (title/abstract/keywords)	Date range	Language
PubMed	(Telemedicine OR Telehealth OR Telecare OR Telemonitoring OR “Remote monitoring” OR “Remote assessments” OR “Remote management” OR “Remote monitoring system” OR “Remote care” OR “Patient telemonitoring” OR “Remote patient monitoring” OR “Self‐monitoring” OR Monitoring OR MHealth OR “Mobile apps” OR “MHealth applications”) AND (Stroke OR “Post‐stroke” OR Poststroke OR “Stroke care” OR “Secondary stroke after stroke”)	01/01/2000–31/10/2023	English
Scopus	TITLE‐ABS‐KEY ((Telemedicine OR Telehealth OR Telecare OR Telemonitoring OR “Remote monitoring” OR “Remote assessments” OR “Remote management” OR “Remote monitoring system” OR “Remote care” OR “Patient telemonitoring” OR “Remote patient monitoring” OR “Self‐monitoring” OR Monitoring OR MHealth OR “Mobile apps” OR “MHealth applications”) AND (Stroke OR “Post‐stroke” OR Poststroke OR “Stroke care” OR “Secondary stroke after stroke”))	01/01/2000–31/10/2023	English
Web of Science	TS = ((Telemedicine OR Telehealth OR Telecare OR Telemonitoring OR “Remote monitoring” OR “Remote assessments” OR “Remote management” OR “Remote monitoring system” OR “Remote care” OR “Patient telemonitoring” OR “Remote patient monitoring” OR “Self‐monitoring” OR Monitoring OR MHealth OR “Mobile apps” OR “MHealth applications”) AND (Stroke OR “Post‐stroke” OR Poststroke OR “Stroke care” OR “Secondary stroke after stroke”))	01/01/2000–31/10/2023	English
Cochrane Library	(Telemedicine OR Telehealth OR Telecare OR Telemonitoring OR “Remote monitoring” OR “Remote assessments” OR “Remote management” OR “Remote monitoring system” OR “Remote care” OR “Patient telemonitoring” OR “Remote patient monitoring” OR “Self‐monitoring” OR Monitoring OR MHealth OR “Mobile apps” OR “MHealth applications”) AND (Stroke OR “Post‐stroke” OR Poststroke OR “Stroke care” OR “Secondary stroke after stroke”)	01/01/2000–31/10/2023	English
ProQuest	(Telemedicine OR Telehealth OR Telecare OR Telemonitoring OR “Remote monitoring” OR “Remote assessments” OR “Remote management” OR “Remote monitoring system” OR “Remote care” OR “Patient telemonitoring” OR “Remote patient monitoring” OR “Self‐monitoring” OR Monitoring OR MHealth OR “Mobile apps” OR “MHealth applications”) AND (Stroke OR “Post‐stroke” OR Poststroke OR “Stroke care” OR “Secondary stroke after stroke”)	01/01/2000–31/10/2023	English
Wiley	(Telemedicine OR Telehealth OR Telecare OR Telemonitoring OR “Remote monitoring” OR “Remote assessments” OR “Remote management” OR “Remote monitoring system” OR “Remote care” OR “Patient telemonitoring” OR “Remote patient monitoring” OR “Self‐monitoring” OR Monitoring OR MHealth OR “Mobile apps” OR “MHealth applications”) AND (Stroke OR “Post‐stroke” OR Poststroke OR “Stroke care” OR “Secondary stroke after stroke”)	01/01/2000–31/10/2023	English
IEEE	(Telemedicine OR Telehealth OR Telecare OR Telemonitoring OR “Remote monitoring” OR “Remote assessments” OR “Remote management” OR “Remote monitoring system” OR “Remote care” OR “Patient telemonitoring” OR “Remote patient monitoring” OR “Self‐monitoring” OR Monitoring OR MHealth OR “Mobile apps” OR “MHealth applications”) AND (Stroke OR “Post‐stroke” OR Poststroke OR “Stroke care” OR “Secondary stroke after stroke”)	01/01/2000–31/10/2023	English

## Appendix 2 (Final Checklist)

Part 1: Demographic Data

How necessary is each of the following demographic data in the system?

**Table:**

Category	Data elements	Very little	Little	Moderate	High	Very high
Demographic information	First Name, last name					
	Father's name					
	National Code					
	Gender					
	Age					
	Weight					
	Height					
	BMI					
	RH (Rhesus) blood type					
	Blood group type					
	Marital status					
	Level of Education					
	Phone number					
	Address					
	Number of family members					
	Occupation					
	Insurance status					
	First name, last name of provider					
	Age of provider					
	Gender of provider					
	Level of Education of provider					
	Specialized field of provider					

Part 2: Patient Clinical Data

How necessary is each of the following Patient Clinical Data of system?

**Table:**

Category	Data Elements	Very little	Little	Moderate	High	Very high
Clinical information of patient medical history	Past medical history					
	Patient symptoms (paresis, dizziness, blurred vision, swallowing disorder)					
	History of drug use and current medications					
	Smoking status					
	Alcohol consumption status					
Clinical information of vital sign	Blood pressure					
	Heart rate					
	Body temperature					
	Respiratory rate					
Clinical information of patient stroke data	Type of stroke (ischemic, hemorrhagic)					
	Date of stroke occurrence					
	Location of brain lesion					
	History of NIHSS score					
	Number of strokes					
	Family history of stroke					
Clinical information of paraclinical data	CBC					
	Lipid profile					
	Vitamin D3					
	Fasting blood sugar					
	CT scan					
	MRI					
	Sonography					
Clinical information of treatment procedure	Drug therapy					
	Rehabilitation treatment (occupational therapy, physiotherapy)					
	Surgical treatment (angiography, angioplasty, etc.)					

Part 3: Functionality of system

How necessary is each of the following functionality of system?

**Table:**

Category	Data elements	Very little	Little	Moderate	High	Very high
Functionality of system	Displaying reports of clinical data					
	Providing alerts for abnormal clinical and vital signs (blood pressure, temperature, heart rate, respiration, weight)					
	Medication reminder					
	Reminder for doing laboratory tests, follow‐up visits, and rehabilitation activities					
	Virtual communication with a doctor (chat forum)					
	virtual communication with a doctor (video call)					
	Responding to frequently asked questions by patients					
	Patient status dashboard					
	Evaluation of the progress of patient					
	Patient education: basic information about stroke (definition and symptoms)					
	Patient education: information about the treatment of stroke					
	Patient education: information about the complications of stroke					
	Patient education: drug information and the importance of medication use					
	Patient education: information about blood pressure and its importance in control					
	Patient education: information about preventing recurrent stroke					
	Patient education: information about poststroke depression and the importance of its treatment					
	Patient education: information about nutrition					
	Patient education: information about rehabilitation and exercise					
	Patient education: information about risk factors					
	Patient education: information about immediate actions in emergency situations					
